# MicroRNA-505 functions as a tumor suppressor in endometrial cancer by targeting TGF-α

**DOI:** 10.1186/s12943-016-0496-4

**Published:** 2016-02-02

**Authors:** Shuo Chen, Kai-Xuan Sun, Bo-Liang Liu, Zhi-Hong Zong, Yang Zhao

**Affiliations:** Department of Gynecology, The First Affiliated Hospital of China Medical University, Shenyang, 110001 China; Department of Biochemistry and Molecular Biology, College of Basic Medicine, China Medical University, Shenyang, 100013 China

**Keywords:** MiR-505, TGF-α, Endometrial carcinoma, Tumorigenesis, Progression

## Abstract

**Background:**

Endometrial carcinoma (EC) is one of the most lethal gynecologic cancers. Patients frequently have regional or distant metastasis at diagnosis. MicroRNAs are small non-coding RNAs that participate in numerous biological processes. Recent studies have demonstrated that miR-505 is associated with several types of cancer; however, the expression and function of miR-505 have not been investigated in EC.

**Methods:**

miR-505 expression in normal endometrial tissue, endometrial carcinomas were quantified by Quantitative reverse transcription PCR. The endometrial carcinoma cell lines HEC-1B and Ishikawa were each transfected with miR-505 or scrambled mimics, after which cell phenotype and expression of relevant molecules were assayed. Dual-luciferase reporter assay and a xenograft mouse model were used to examine miR-505 and its target gene TGF-α.

**Results:**

RT-PCR results demonstrated that miR-505 was significantly downregulated in human EC tissues compared to normal endometrial tissues. Besides, miR-505 expression was negatively associated with FIGO stage (stage I-II vs. III-IV), and lymph node metastasis (negative vs. positive). In vitro, overexpression of miR-505 significantly suppressed EC cell proliferation, increased apoptosis and reduced migratory and invasive activity. A miR-505 binding site was identified in the 3′ untranslated region of TGF-α mRNA (*TGFA*) using miRNA target-detecting software; a dual luciferase reporter assay confirmed that miR-505 directly targets and regulates *TGFA*. RT-PCR and Western-blotting results indicated that overexpressing miR-505 reduced the expression of TGF-α and the TGF-α-regulated proteins MMP2, MMP9, CDK2, while induced Bax and cleaved-PARP expression in EC cells. In vivo, overexpression of miR-505 reduced the tumorigenicity and inhibited the growth of xenograft tumors in a mouse model of EC.

**Conclusions:**

Taken together, this study demonstrates that miR-505 acts as tumor suppressor in EC by regulating TGF-α.

**Electronic supplementary material:**

The online version of this article (doi:10.1186/s12943-016-0496-4) contains supplementary material, which is available to authorized users.

## Background

Endometrial carcinoma (EC) is one of the three most common types of gynecologic cancer and its global incidence has increased in recent years [1]. It is known that endometrial carcinoma is divided into estrogen dependent (type I) and non-estrogen dependent (type II) [2, 3]. The pathogenesis of type I endometrial carcinoma is that under the long-term effects of estrogen, but without the antagonistic effects of progesterone, the endometrium progressively increases in thickness and therefore becomes more susceptible to becoming cancerous [4, 5]. Generally, FIGO stage I (2009) endometrial carcinoma is considered to have a good prognosis with a 5-year survival rate of up to 96 % [6]. Unfortunately, as multiple factors affect recurrence, including surgical stage, differentiation and lymph node metastasis, effective therapies for patients with advanced-stage EC or disease recurrence are still lacking [7], the 5-year OS rate of the FIGO stages II–IV was 76.0 % [8]. The identification and further elucidation of the molecular mechanisms responsible for EC tumorigenesis and progression may have a major impact on the health of females.

MicroRNAs are a group of endogenous, small, 18–25 base-long non-coding RNAs that influence multiple biological processes including proliferation, apoptosis, senescence, differentiation and development by post-transcriptionally repressing gene expression or inducing mRNA degradation via binding to the 3′ untranslated regions (UTR) of specific target genes [9–11]. MicroRNAs can target approximately 20–30 % of genes. A single microRNA can target at least 200 genes, and a single gene can be regulated by many RNAs [12–14]. A large body of research has demonstrated that dysregulation of microRNAs can promote tumorigenesis and metastasis [15, 16]. Recent studies have demonstrated that microRNAs can act as either potent oncogenes or tumor suppressor genes [17].

MiR-505 has been identified to function as a tumor suppressor in breast cancer [18–20]. Numerous studies have indicated that miR-505 inhibits cell proliferation by inducing apoptosis and can promote chemoresistance in breast cancer [18, 21]. Expression of miR-505 correlates with established prognostic biomarkers in breast cancer [22]. Moreover, data from mouse models has indicated an association between miR-505 and human basal-type breast cancer [19]. Another study recently provided evidence that miR-505 could suppress the epithelial-mesenchymal transition (EMT) and metastasis in nasopharyngeal carcinoma [23]. However, little is known about the biological function and target genes of miR-505 in EC.

Transforming growth factor-α (TGF-α) is a member of the epidermal growth factor family of mitogens and is encoded by the *TGFA* gene. TGF-α binds to the epidermal growth factor receptor (EGFR) to initiate a series of biological processes including cell proliferation, differentiation and development [24]. TGF-α makes an important contribution to cell proliferation and invasion in triple-negative breast cancer [25], and strongly correlates with metastasis in advanced prostate cancer [26]. Besides, TGF-α is also involved in EC tumorigenesis and reported to promote angiogenesis in endothelial cells [27, 28]. Zhu et al. reported that miR-152 could potentially controls migration and invasion by targeting TGF-α in prostate cancer cell lines [29]; Jin et al. reported that miR-376c could inhibit cell proliferation and invasion by targeting TGF-α in osteosarcoma [30]; Qin et al. reported that miR-124 may regulates TGF-α-induced EMT in human prostate cancer cells [31]. These studies suggest us that TGF-a may have potential as a target in inhibiting endometrial cancer tumorigenesis and progression through down-regulating by relative microRNA. We analyzed the mRNA sequence of TGF-α and identified binding sites for miR-505 in *TGFA*.

Here, we report that miR-505 functions as a tumor suppressor in EC by targeting and regulating *TGFA*.

## Results

### Associations between miR-505 expression and the clinicopathological features of EC

**Fig. 1:**
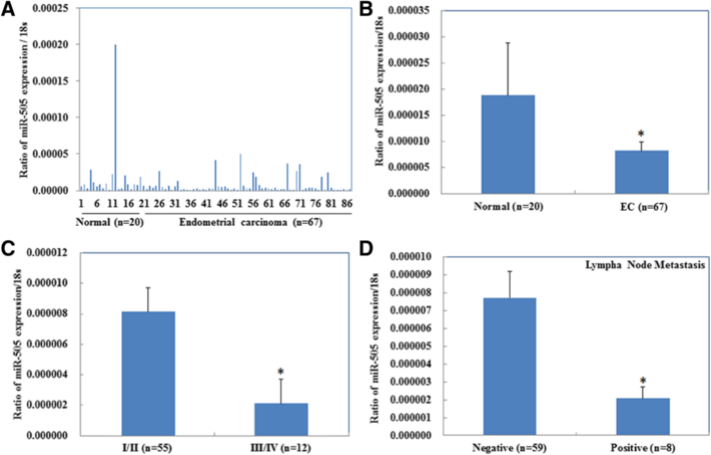
Associations between miR-505 expression and the clinicopathological features of EC. miR-505 mRNA expression was significantly lower in endometrial adenocarcinoma than in normal endometrial tissues (**a**-**b**), and was negatively associated with FIGO stages (**c**), and Lymph node metastasis (**d**). EC = endometrial adenocarcinoma, *P < 0.05

Quantitative reverse transcription polymerase chain reaction (qRT-PCR) demonstrated that miR-505 was expressed at lower levels in human EC than normal endometrial tissues (Fig. 1a, b, *P* < 0.05, Additional file 1: Table S1). Besides, miR-505 expression was negatively associated with FIGO stage (Fig. 1c, stage I/II vs. stage III/IV, *P* < 0.05), and lymph node metastasis (Fig. 1d, negative vs. positive, *P* < 0.05). There were no significant associations between miR-505 expression and the depth of invasion and pathological type (endometrial adenocarcinoma vs. other types, *P* > 0.05, Details could be found in Additional file 1: Table S2).

### Overexpression of miR-505 inhibits proliferation, induces apoptosis and reduces the tumorigenicity of EC cells in vitro

**Fig. 2:**
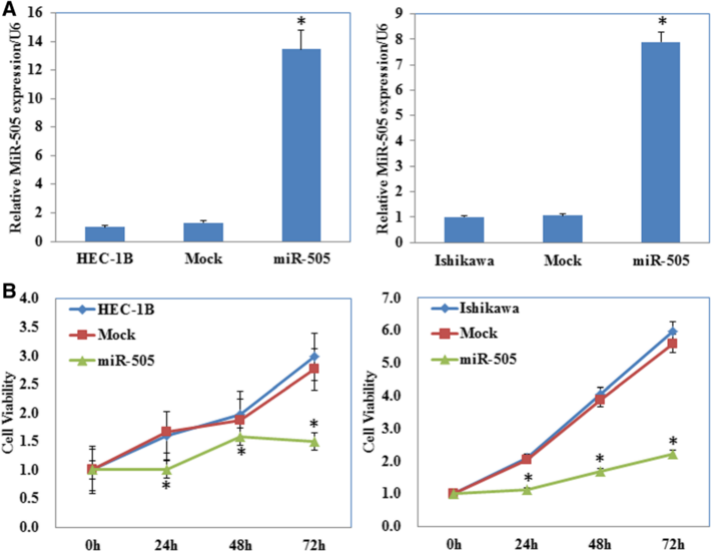
miR-505 overexpression suppresses endometrial carcinoma cell proliferation in vitro. miR-505 transfection exhibited significantly higher miR-505 expression (**a**) and slower growth ability (**b**) compared with the control and mock cells. Results are representative of three separate experiments; data are expressed as the mean ± standard deviation, *P < 0.05

**Fig. 3:**
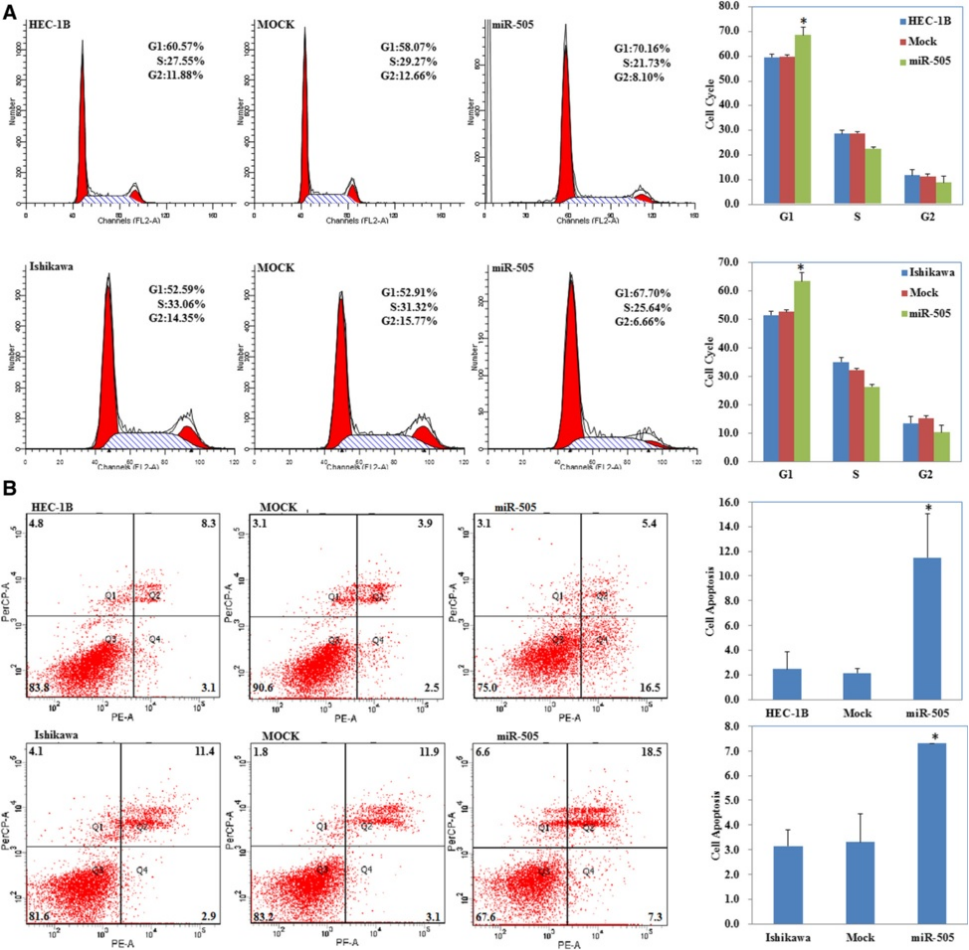
miR-505 overexpression induces G1 phase arrest and promotes apoptosis of endometrial carcinoma cells. MiR-505 transfection significantly induced G1 arrest (**a**) and elevated apoptosis (**b**) compared with the control and mock cells. Results are representative of three separate experiments; data are expressed as the mean ± standard deviation, *P < 0.05

**Fig. 4:**
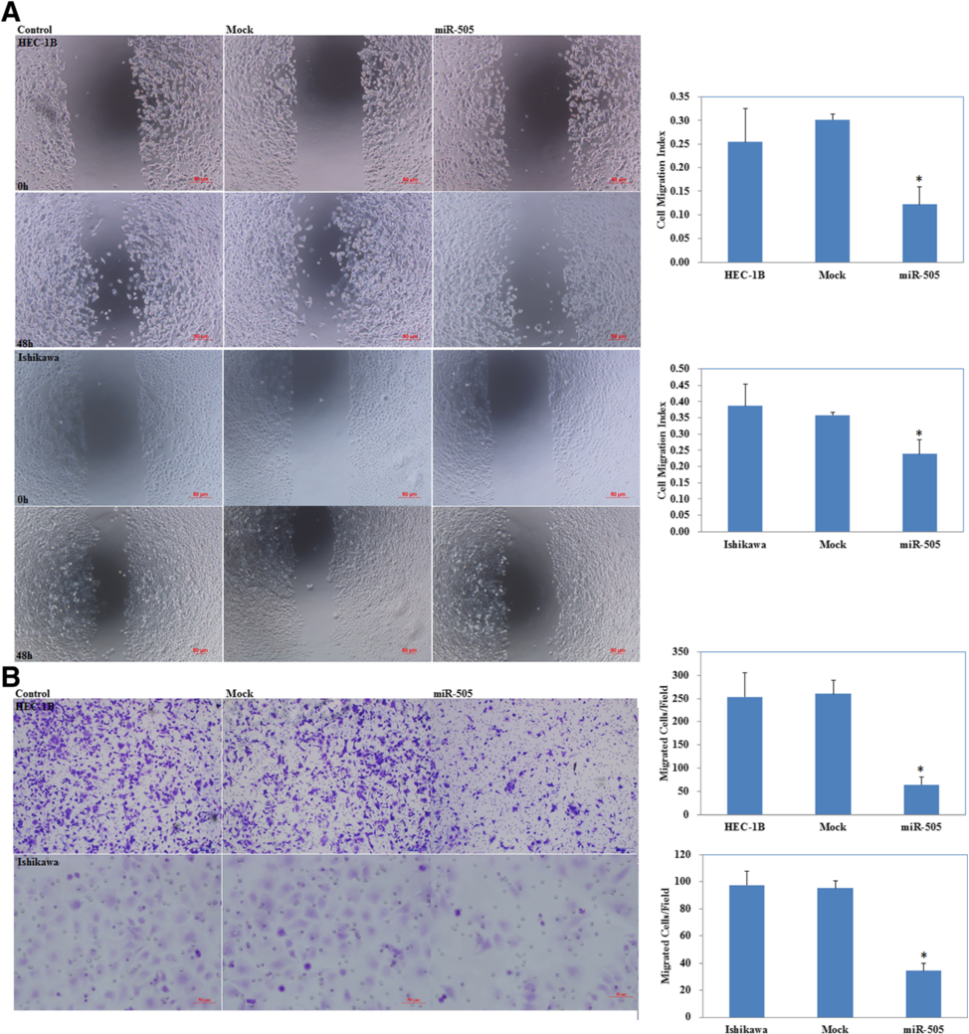
Effects of miR-505 transfection on invasive and metastatic ability of endometrial adenocarcinoma cell lines in vitro. miR-505 overexpression reduced cell migration (**a**), and invasion ability (**b**) compared with the control and mock cells. Results are representative of three separate experiments; data are expressed as the mean ± standard deviation, *P < 0.05

Ishikawa and HEC-1B cell lines were transfected with miR-505 mimic or the scrambled mimic. The CCK-8 assay demonstrated that the miR-505 overexpression (Fig. 2a, *P* < 0.05) significantly inhibited cell proliferation in both cell lines compared with the mock or negative control group (Fig. 2b, *P* < 0.05). Furthermore, cytometry flow showed that overexpression of miR-505 induced significant G1 phase arrest (Fig. 3a, *P* < 0.05; PI staining) and high levels of apoptosis (Fig. 3b, *P* < 0.05, Annexin V-FITC and PI staining). Additionally, overexpression of miR-505 significantly reduced the invasion (Fig. 4a, *P* < 0.05, Transwell assay) and migratory ability of both cell lines (Fig. 4b, *P* < 0.05, wound healing assay). Details could be found in Additional file 1: Table S3.

### miR-505 directly targets TGFA and regulates TGF-α expression in EC cells

**Fig. 5:**
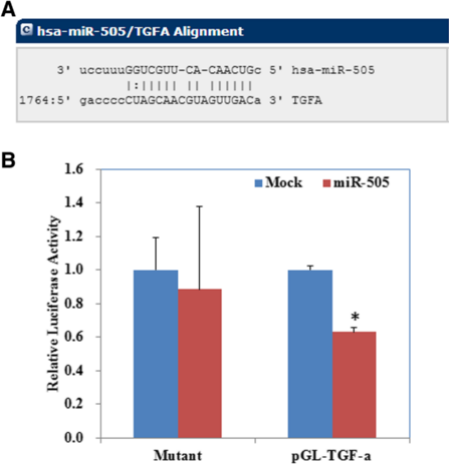
miR-505 directly targets TGFA. The predicted seed region in the 3′ UTR of TGF-α (**a**); Luciferase activity is unchanged when using a scrambled miRNA sequence or miR-505 with the mutant 3′-UTR of TGF-α. However, when the wild-type 3′-UTR of TGF-α is used, the promoter activity is significantly reduced by miR-505 (**b**). Results are representative of three separate experiments; data are expressed as the mean ± standard deviation, *P < 0.05

**Fig. 6:**
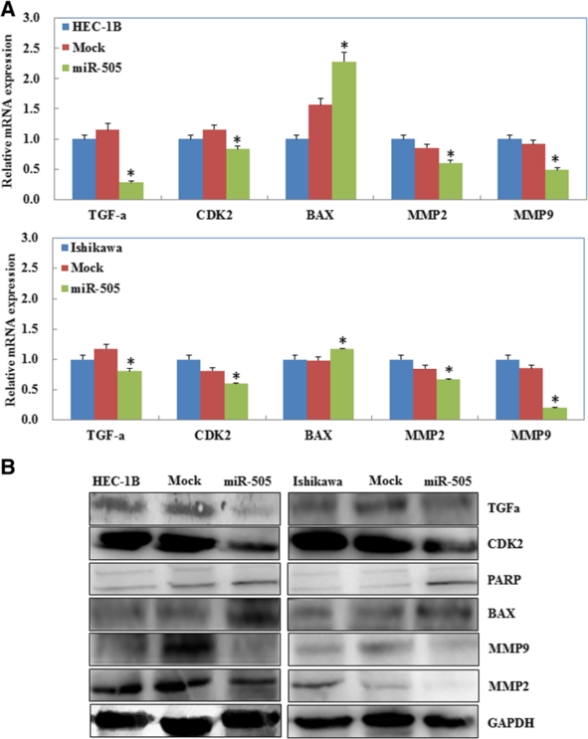
Effects of miR-505 transfection on endometrial adenocarcinoma cell genotype in vitro. miR-505 overexpression reduced TGF-α, CDK2, MMP2 and MMP9 expression, while increased cleaved PARP and Bax mRNA (**a**) or protein (**b**) expression. *P < 0.05

We analyzed the mRNA sequence of TGF-α using the miRNA target-detecting software and identified binding sites for miR-505 in *TGFA* (Fig. 5a). A dual-luciferase reporter assay indicated that miR-505 can bind to the 3′ UTR of *TGFA* (Fig. 5b). Furthermore, RT-PCR and Western blotting demonstrated that overexpression of miR-505 downregulated the mRNA (Fig. 6a, *P* < 0.05) or protein (Fig. 6b, *P* < 0.05) expression of MMP2, MMP9, CDK2, and TGF-α, while upregulated Bax and cleaved PARP expression in both cells.

### Overexpression of miR-505 inhibits the growth of EC xenografts in a mouse model

**Fig. 7:**
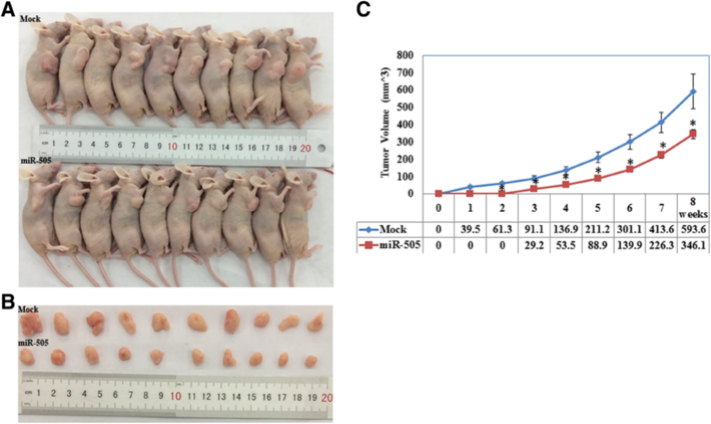
Overexpression of miR-505 inhibits the growth of tumor xenografts in a mouse model. Tumor xenograft volume in nude mice treated with miR-505 was smaller than that in mock nude mice (**a** and **b**). The growth rate was also slower than that in the mock group (**c**). *P < 0.05

**Fig. 8:**
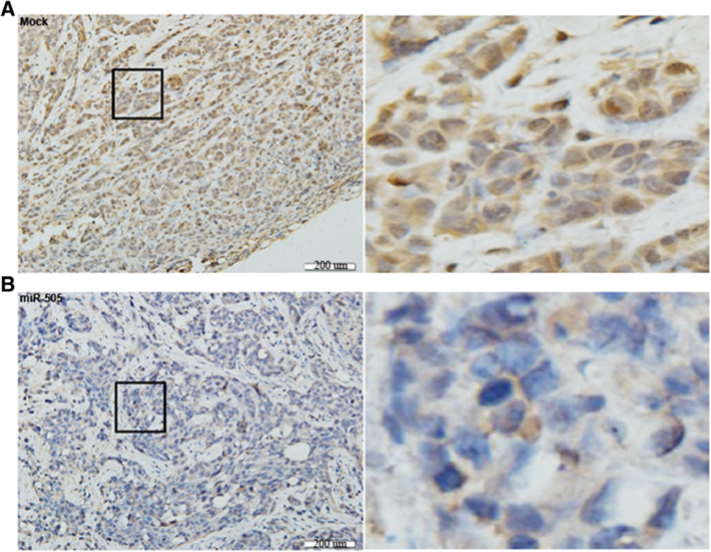
Overexpression of miR-505 inhibits TGF-α expression in vivo. Immunohistochemical analysis demonstrated a significant reduction of TGF-α expression in the HSA-miR-505 group (**b**) compared with the mock group (**a**) in nude mice tumor tissues

We stably transfected HEC-1B cells with has-miR-505 or negative control vector (Mock) and established a mouse model of EC. Compared to the mock group, HEC-1B cells overexpressing miR-505 had a significantly lower tumor volume (Fig. 7a and b, *P* < 0.05) and slower rate of growth (Fig. 7c, *P* < 0.05). To determine whether miR-505 affected the expression of TGF-α in vivo, we performed immunohistochemical staining of the excised xenograft tumors. The tumors formed by the cells overexpressing miR-505 had a significantly lower TGF-α expression compared to the mock group (Fig. 8a and b).

## Discussion

This study demonstrates that miR-505 is significantly downregulated in human EC compared to normal endometrial tissues, and the expression of miR-505 was significantly associated with the FIGO stage and lymph node metastasis. Additionally, overexpression of miR-505 significantly inhibited EC cell proliferation, induced G1-S arrest and apoptosis, and suppressed EC cell invasion and migration in vitro. Taken together, this data indicates that downregulation of miR-505 could promote the development and progression of EC.

The growth factor TGF-α is mainly secreted during embryogenesis and is frequently overexpressed in malignant tumors, including osteosarcoma, hepatic cancer, prostate cancer and breast cancer [20, 22, 26, 32, 33]. Recent studies have linked TGF-α overexpression to the invasive ability of triple-negative breast cancer cells [34]. Interestingly, overexpression of TGF-α could interact with mesenchymal-to-epithelial transition (MET) and contribute to cetuximab resistance in patients with colorectal cancer [35, 36]. High TGF-α expression was prognostic for poor overall survival in high-risk patients with melanoma [37]. In this study, target prediction software identified a miR-505 binding site in the 3′ UTR of *TGFA*, and dual-luciferase reporter assays confirmed that *TGFA* is a direct target of miR-505. In confirmation of this functional interaction, overexpression of miR-505 reduced both TGF-α mRNA and protein expression in EC cell lines.

TGF-α binds to the epidermal growth factor receptor (EGFR) to initiate multiple cellular events, including cell proliferation, and this process has been associated with tumorigenesis and angiogenesis [38, 39]. We infer that upregulation of miR-505 may inhibit proliferation, migration and invasion; promote apoptosis in EC by leading to decreased expression of TGF-α. MMP2 and MMP9 are two of the most studied members of the matrix metalloproteinase (MMPs) family, and play central roles in cell invasion and metastasis by cleaving components of the extracellular matrix (ECM) [27]. In this study, overexpression of miR-505 downregulated both MMP2 and MMP9 mRNA and protein expression, providing further evidence that upregulation of miR-505 may inhibit invasion and metastasis in EC.

During late G1 phase, the CDK2/cyclin E complexes regulate the G1 to S phase transition and the CDK2/cyclin A complexes play an important role in S phase progression [40, 41]. Bcl-2 associated X protein (Bax) is a representative member of Bcl-2 family that promotes apoptosis via the caspase-dependent pathway and is frequently downregulated in cancer [42]. Poly ADP-ribose polymerase (PARP) participates in DNA repair and can inhibit apoptosis [43–45]. In this study, overexpression of miR-505 reduced CDK2 mRNA and protein expression, upregulated Bax and increased the levels of cleaved PARP. Therefore, these results indicate that upregulation of miR-505 promotes apoptosis in EC by upregulating Bax and PARP and inducing G1-S stage arrest via downregulation CDK2.

A mouse model of EC was established to further investigate the function of miR-505 in EC in vivo. Overexpression of miR-505 significantly inhibited tumor xenograft growth and led to a reduced tumor volume. IHC staining confirmed that the tumors formed by miR-505-transfected cells expressed lower levels of TGF-α. These results provide further confirmation that miR-505 might act as tumor suppressor in EC by targeting *TGFA*.

## Conclusion

In conclusion, we demonstrate that miR-505 suppresses cell proliferation, invasion and metastasis in EC by targeting *TGFA*. This knowledge may provide the basis for further investigations to identify novel diagnostic and therapeutic methods for EC.

## Methods

### Tissue specimen collection

67 Endometrial adenocarcinomas (ECs) and 20 normal endometrial specimens were collected from patients who underwent surgical resection at the Gynecology Department, First Affiliated Hospital of China Medical University (Shenyang, Liaoning Province, China) between May 2013 and May 2015. No patients had received any treatment before surgery. Tumor staging and pathology were based on the FIGO criteria (2009). The China Medical University ethic committee approved this research project and informed consent was obtained from each patient. Tissues were immediately frozen in liquid nitrogen and stored at −80 °C until analysis.

### Cell culture and transfection

The human endometrial cancer cell lines HEC-1B (poorly differentiated, estrogen receptor-negative endometrial adenocarcinoma) and Ishikawa (highly differentiated, estrogen receptor-positive endometrial adenocarcinoma) were obtained from the ATCC (Manassas, VA, USA) and maintained in Dulbecco’s Modified Eagle’s Medium (DMEM) containing 10 % fetal bovine serum and 1 % penicillin and streptomycin at 37 °C in a humidified atmosphere containing 5 % CO_2_. Cells were each transfected with miR-505 mimic using Lipofectamine 2000 reagent (Invitrogen, Carlsbad, USA) according to the manufacturer’s protocol. The miR-505 mimic sequence was GGG AGC CAG GAA GUA UUG AUG U, and the scrambled mimic (Mock) sequence was ACU ACU GAG UGA CAG UAG A. The scrambled mimic was also transfected in cells as negative control.

### Cell proliferation assay

Cell proliferation was monitored using CCK-8 reagent (Dojindo, Tokyo, Japan) following the manufacturer’s protocol. EC cells were transfected with miR-505 mimic or scrambled mimic, seeded into 96-well plates (2 × 10^3^ cells per well) and incubated until adherent. Cell proliferation was assessed every 24 h (up to 72 h); 10 μL CCK-8 solution was added to the medium in each well, incubated for 4 h and the absorbance values were measured at 450 nm using a microplate reader.

### Cell cycle analysis

Cells were trypsinized, harvested, washed twice with PBS, fixed with 70 % ice-cold ethanol in −20 °C overnight, washed with PBS, and then incubated with RNAase and stained with PI (BD Biosciences, New Jersey, USA) following the manufacturer’s protocol. The PI signal was examined by a flow cytometry; a total of 10,000 cells were assessed for each sample.

### Apoptosis assay

Apoptosis was quantified using 7AAD and PE-labeled Annexin V (BD Biosciences) following the manufacturer’s protocol and flow cytometry. Cells were collected 48 h after transfection, washed twice with cold PBS, and resuspended at 1 × 10^6^ cells/mL and mixed with 100 μL of 1× buffer and 5 μL Annexin V- PE and 7AAD, incubated for 15 min in the dark, 400 μL 1× buffer was added, and the cells were subjected to cytometry flow within 1 h.

### Wound healing assay

Cells were seeded into 6-well plates, allowed to adhere and transfected with miR-505 mimic or scrambled mimic, linear wounds were created in the confluent monolayers using a pipette tip. Cells were washed three times with PBS, the medium was changed to FBS-free DMEM, and photos were captured using a light microscope at 0 h, 24 h and 48 h. The wound healing rate = (Area of original wound − Area of actual wound at different times)/Area of original wound × 100 %.

### In vitro cell invasion assay

Cells transfected with miR-505 mimic or mock mimic were collected after 48 h, re-suspended in serum-free DMEM medium, and 5 × 10^4^ cells in serum-free medium were seeded into the upper chamber of Transwell inserts that had been pre-coated with Matrigel (40 μL of 8 mg/mL stock solution; Becton-Dickinson Labware, Bedford, MA, USA); normal culture medium was placed into the lower chamber. Cells were allowed to migrate for 48 h at 37 °C, then the membranes were fixed with methanol and stained with 0.1 % crystal violent. Cell invasion was measured by counting the number of cells attached to the lower side of the membrane in ten high-powered (200×) fields under a light microscope.

### Quantitative RT-PCR

Total RNA was isolated from the treated cells and human tissues using TRIzol reagent (Takara, Shiga, Japan) according to the manufacturer’s instructions, after which OD260/280/320 was measured by spectrophotometer (Unico, Shanghai, China). The value of OD260/280 within 1.8–2.0 means high RNA quality, and the sample concentration = sample dilution factor * (OD260-OD320) * 0.04. 2 μg was reverse-transcribed to complementary DNA (cDNA) using avian myeloblastosis virus transcriptase and random primers (Takara). Real-time RT-PCR analysis was performed in triplicate on the ABI prism 7000 sequence detection system (Applied Biosystems) using the SYBR Green PCR Master Mix (Applied Biosystems, Eugene, OR, USA). The relative expression of target genes was determined by comparing the threshold cycle (Ct) of the target genes to the Ct of 18S rRNA (*18 s*) using the 2^-ΔΔCT^ method [46]. Hairpin-it™ microRNA and U6 snRNA Normalization RT-PCR Quantitation (GenePharma) was used to check mature miR-505 in cells.

### Western blotting

Cells and tissues were lysed in ice-cold RIPA lysis buffer and the concentrations were measured using the protein assay kit (Bio-Rad Laboratories, Hercules, CA, USA). Protein samples (60 μg) were separated by SDS-PAGE on 10 % gels and transferred onto Hybond membranes (Amersham, Munich, Germany). After blocking with 5 % fat-free milk for 1 h, the membranes were incubated with primary antibodies against MMP2, MMP9, Cyclin A1, CDK2, Bax, PARP, TGF-α (1:500, Proteintech, Proteintech Group, USA) and GAPDH (1:2000, Proteintech, Proteintech Group, USA) overnight in 4 °C, then incubated with anti-mouse, anti-rabbit or anti-goat IgG secondary antibodies (1:5000; Dako, Carpinteria, CA, USA) for 1 h. The bands were developed using ECL Plus detection reagent (Santa Cruz Biotechnology) and visualized on X-ray film (Fujifilm, Tokyo, Japan) using Image Quant LAS 4000 (Fujifilm). Band densities were determined and expressed relative to the internal control GAPDH.

### Dual-luciferase reporter assay

Luciferase assays were carried out to confirm the interaction between miR-505 and the 3′ untranslated region (UTR) of *TGFA*. HEK293T cells were seeded into 24-well plates, cotransfected with 50 nM miR-505 or scrambled mimic and 600 ng of dual luciferase vector expressing the wild-type or mutant 3′-UTR TGF-α sequences. After 24 h, the luciferase activities were measured using the Dual-Luciferase Reporter Assay System (Promega, Madison, WI, USA) according to the manufacturer’s protocol. The ratio of firefly to renilla luciferase signal was used to normalize firefly activity for intra-experimental transfection efficiency.

### In vivo tumorigenesis model

An in vivo model of EC was established by subcutaneously injecting 5-week-old female BALB/c nude mice with 1 × 10^7^ HEC-1B cells transfected with miR-505 (or mock transfected) suspended in PBS (10 mice per group). The weight of the mice and tumor volumes were determined every 3 days; tumor volume was assessed by measuring the length (*L*) and width (*W*) using calipers (tumor volume, mm^3^ = 0.5 × L× W^2^. After 8 weeks, the mice were euthanized and the tumors were excised, measured and photographed. All mice were obtained from Shanghai SLAC Laboratory Animal, Co., Ltd. (Shanghai, China) and housed in a specific pathogen-free environment. This experiment was in accordance with the National Institutes of Health Guide for the Care and Use of Laboratory Animals and approved by China Medical University Animal Care and Use Committee.

### Immunohistochemistry

Consecutive tissue sections of the xenograft tumors were subjected to IHC to detect TGF-α expression. After deparaffinization, dehydration and boiling to unmask antigens, sections were blocked and incubated with TGF-α antibodies overnight at 4 °C, then incubated with HRP-conjugated anti-rabbit antibodies (Dako) for 15 min.

### Statistical analysis

Data are presented as mean ± SD and were analyzed using SPSS 17.0 software (SPSS Inc., Chicago, IL, USA). The unpaired two-tailed Student’s *t*-test, Mann–Whitney *U*-test and Spearman’s correlation test were used to compare the two groups. All *P*-values are two-sided; *P* < 0.05 represents statistical significance.

## Additional file

Additional file 1: Table S1. miR-505 expression in normal endometrial and endometrial. **Table S2.** Correlation of miR-505 expression with different clinicopathological features of endometrial carcinoma. **Table S3.** Cell phenomenon studies. (DOCX 24 kb)
