# Supplementary material for: MicroRNA-505 functions as a tumor suppressor in endometrial cancer by targeting TGF-α
Source: Mol Cancer. 2016 Feb 2;15:11. doi: 10.1186/s12943-016-0496-4 (PMC4736705; doi:10.1186/s12943-016-0496-4)
Supplement: Additional file 1: Table S1. — miR-505 expression in normal endometrial and endometrial. Table S2. Correlation of miR-505 expression with different clinicopathological features of endometrial carcinoma. Table S3. Cell phenomenon studies. (DOCX 24 kb) [file 12943_2016_496_MOESM1_ESM.docx]

**Supplementary Table 1:** miR-505 expression in normal endometrial and endometrial carcinoma tissues

| **Groups** | **N** | **miR-505 expression / 18s** | ***P* value** |
| --- | --- | --- | --- |
|  |  |  |  |
| Normal endometrial | 20 | 0.0000189 ± 0.0000445 | ***0.05*** |
| Endometrial carcinoma | 67 | 0.00000705 ± 0.0000108 |  |

The fold-difference of control samples to endometrial carcinoma was 2.68.

Bold and Italics means P < 0.05.

**Supplementary Table 2:** Correlation of miR-505 expression with different clinicopathological features of endometrial carcinoma

| **Clinicopathological features** | | **N** | **miR-505 expression / 18s** | ***P* value** |
| --- | --- | --- | --- | --- |
|  |  |  |  |  |
| **The pathology types** | |  |  | 0.88 |
| Endometrioid adenocarcinoma | | 54 | 7.14E-06 ± 0.0000115 |  |
| The other pathology types | | 13 | 6.65E-06 ± 6.61E-06 |  |
| **Age** | |  |  | 0.09 |
| <55 | | 30 | 4.53E-06 ± 7.43E-06 |  |
| ≥ 55 | | 37 | 9.08E-06 ± 0.0000124 |  |
| **FIGO stages** | |  |  | ***0.04*** |
| I-II | | 55 | 8.12E-06 ± 0.0000116 |  |
| III-IV | | 12 | 2.14E-06 ± 2.20E-06 |  |
| **Pathology classification** | |  |  | 0.68 |
| Well | | 35 | 6.13E-06 ± 9.09E-06 |  |
| Moderate | | 14 | 9.34E-06 ± 0.0000133 |  |
| Poor | | 18 | 7.04E-06 ± 0.0000110 |  |
| **The depth of myometrial infiltration** | | |  | 0.60 |
| < 1/2 | 37 | | 7.68E-06 ± 0.0000110 |  |
| ≥ 1/2 | 30 | | 6.27E-06 ± 0.0000102 |  |
| **Lymph node metastasis** |  | |  | ***0.00*** |
| Negative | 59 | | 7.72E-06 ± 0.0000112 |  |
| Positive | 8 | | 2.07E-06 ± 1.82E-06 |  |
| Bold and Italics means P < 0.05. | | | | |

**Supplementary Table 3: Cell phenomenon studies**

| **Cell Viability** | | | | |
| --- | --- | --- | --- | --- |
|  | 0h | 24h | 48h | 72h |
| HEC-1B | 0.1089 ± 0.0047 | 0.1742 ± 0.0073 | 0.2145 ± 0.0144 | 0.3245 ± 0.0072 |
| Mock | 0.1123 ± 0.0038 | 0.1869 ± 0.0083 | 0.2107 ± 0.0029 | 0.3103 ± 0.0005 |
| miR-505 | 0.1024 ± 0.0029 | 0.1034 ± 0.0072 | 0.1618 ± 0.0041 | 0.1536 ± 0.0067 |
|  | 0h | 24h | 48h | 72h |
| Ishikawa | 0.1345 ± 0.0035 | 0.2825 ± 0.0385 | 0.5447 ± 0.0012 | 0.8029 ± 0.0166 |
| Mock | 0.1413 ± 0.0167 | 0.2897 ± 0.0097 | 0.5468 ± 0.0166 | 0.7913 ± 0.0493 |
| miR-505 | 0.1230 ± 0.0083 | 0.1378 ± 0.0135 | 0.2079 ± 0.0267 | 0.2731 ± 0.0340 |
| **Cell Cycle** | | | | |
|  | G1 | S | G2 | P value |
| HEC-1B | 59.49 ± 0.98 | 28.62 ± 1.11 | 11.89 ± 0.44 | ***6.0E-05*** |
| Mock | 59.80 ± 1.55 | 28.76 ± 0.52 | 11.45 ± 1.05 | ***7.1E-05*** |
| MiR-505 | 68.58 ± 2.12 | 22.58 ± 1.97 | 8.83 ± 0.57 |  |
|  | G1 | S | G2 | P value |
| Ishikawa | 51.48 ± 1.44 | 35.03 ± 1.49 | 13.50 ± 2.29 | ***0.002*** |
| Mock | 52.58 ± 0.63 | 32.18 ± 0.62 | 15.24 ± 0.82 | ***0.002*** |
| miR-505 | 63.43 ± 3.10 | 26.32 ± 0.70 | 10.25 ± 2.55 |  |
| **Cell apoptosis rate (%)** | | | | |
|  | HEC-1B | P value | Ishikawa | P value |
| Untreated | 2.48 ± 1.39 | ***0.011*** | 3.15 ± 0.66 | ***2.9E-05*** |
| Mock | 2.15 ± 0.34 | ***0.007*** | 3.30 ± 1.56 | ***4.2E-04*** |
| miR-505 | 11.50 ± 3.54 |  | 7.30 ± 0.08 |  |
| **Wound healing** | | | | |
|  | HEC-1B | P value | Ishikawa | P value |
| Untreated | 0.254 ± 0.072 | ***0.040*** | 0.386 ± 0.067 | ***0.050*** |
| Mock | 0.301 ± 0.012 | ***0.003*** | 0.358 ± 0.094 | ***0.007*** |
| miR-505 | 0.122 ± 0.038 |  | 0.238 ± 0.043 |  |
| **Transwell** | | | | |
|  | HEC-1B | P value | Ishikawa | P value |
| Untreated | 253.33 ± 52.50 | ***0.008*** | 97.67 ± 10.21 | ***0.001*** |
| Mock | 260 ± 29.44 | ***0.001*** | 95.33 ± 5.56 | ***0.000*** |
| miR-505 | 64 ± 16.06 |  | 34.33 ± 5.44 |  |
| Bold and Italics means P < 0.05, miR-505 group vs. the other groups. | | | | |
